# Health status follow-up of migrant children after arrival in France: A multicenter study in hospital outpatient clinics in the Paris region

**DOI:** 10.1016/j.jmh.2026.100412

**Published:** 2026-04-17

**Authors:** Alice Grognet Essaian, Frédéric Sorge, Enora Le Roux, Sophie Guilmin-Crépon, Juliette Goutines, Laurie C. Miller, Marion Taine, Romain Basmaci, Albert Faye

**Affiliations:** a Department of General Paediatrics, Paediatric Infectious Disease and Internal Medicine, AP-HP, Robert-Debré Hospital, Université Paris Cité F-75019 Paris, France; b Department of General Paediatrics and Paediatric Infectious Diseases, AP-HP, Necker-Enfants malades Hospital, Université Paris Cité F-75015 Paris, France; c INSERM 1123, Epidémiologie clinique-évaluation économique appliqué aux populations vulnérables (ECEVE) F-75010 Paris, France; d Clinical Epidemiology Unit, INSERM CIC-EC 1426, APHP, Robert-Debré Hospital F-75019 Paris, France; e Friedman School of Nutrition Science and Policy and Eliot-Pearson Department of Child Study & Human Development, Tufts University, Boston, Massachusetts, USA; f Université Paris Cité, INSERM 1153, Center for Research in Epidemiology and Statistics (CRESS), F-75004 Paris, France; g Department of Emergency and Paediatrics, APHP, Louis-Mourier Hospital F-92700 Colombes, France; h Université Paris Cité, INSERM 1137, Infection, Antimicrobials, Modelling, Evolution (IAME) F-75018 Paris, France

**Keywords:** Migrant children, Health status, Precarity, Anthropometric status, Infections

## Abstract

**Purpose:**

Many studies have examined the health issues of international migrant children upon arrival in their host country. However, very few have described their health status long after settling. We aimed to assess the health status of migrant children two years after their arrival in a high-income country.

**Methods:**

We conducted a descriptive, longitudinal study among migrant children accompanied by parents who attended a migrant outpatient clinic in the Paris region, France, upon arrival between January 2018 and January 2021. A follow-up consultation was offered to all children at least two years after the initial consultation. Only children who attended the follow-up consultation were included in the study, and findings compared to status at initial consultation.

**Results:**

Fifty-seven children were included. The median age at arrival was 5.5 years and the male-to-female ratio was 0.73. The median delay between the two consultations was 2.8 years. The majority of the children (84%) were from Sub-Saharan Africa. At the follow-up consultation, half of the families had unstable housing. Thirty-three children (58%) had not received routine medical care. Overweight prevalence increased from 5% to 19% between the two visits (*p* < 0.01). No cases of early puberty were observed. Two children were diagnosed with delayed psychomotor development at the follow-up visit.

**Conclusion:**

Health issues in migrant children may be exacerbated by persistent precarity for at least two years after arrival. Beyond initial care, new immigrant families require long-term, specific medical and preventive support.

## 1 Introduction

Many studies describe the health status of international migrant children upon arrival in their host country (Baauw et al., 2019; Kadir et al., 2019). Recently settled migrants have been found to be at risk of respiratory infections, viral hepatitis and skin infections, including tinea capitis (Cuétara et al., 1998; Huaume et al., 2017; Lamb and Rademaker, 2001; Mashiah et al., 2016; Pérez-Crespo et al., 2016). Other non-infectious health issues have also been reported in migrant children, including nutritional issues (Gualdi-Russo et al., 2014; Hjern et al., 1991; Jaeger et al., 2012; Labree et al., 2011; Reifen et al., 2003), central early puberty (Gualdi-Russo et al., 2014; Soriano-Guillén et al., 2010; Teilmann et al., 2006), poorer psychomotor development (Greier and Riechelmann, 2014; Lehti et al., 2018; Mor et al., 2018; Muggli et al., 2022), autism spectrum disorders (Crafa and Warfa, 2015; Gao et al., 2022; Morinaga et al., 2021; Schmengler et al., 2021; Schmengler et al., 2019), and increased risk of post-traumatic stress disorder (Almqvist and Brandell-Forsberg, 1997; Deriu et al., 2018; Montgomery, 2022; Reavell and Fazil, 2017; Richter et al., 2020). However, little is known about the longer-term physical health outcomes of these children beyond the initial post-arrival period.

Several paediatric migrant outpatient clinics within hospital tertiary care centres in the Paris region are dedicated to the management and follow-up of recently arrived migrant children from low-income countries, particularly from sub-Saharan Africa. These clinics provide migrant children, who are not covered by health insurance, with free access to care. They offer health screening and catch-up vaccinations, in accordance with current French recommendations (Bilan de santé à réaliser chez toute personne migrante primo-arrivante (adulte et enfant), 2024). Once social coverage has been obtained, and in the absence of specific health issues, children are referred to general practitioners for further routine medical follow-up.

The aim of this study was to describe the evolution of the health status of a cohort of migrant children returning to these specialised hospital outpatient clinics more than 2 years after their arrival in France. Specifically, we aimed to describe changes in these children’s anthropometric status, as well as to assess their psychomotor and pubertal development. Furthermore, we investigated the integration of these children into the French healthcare system and their vaccination coverage.

## 2 Materials and methods

### 2.1 Study design

This multicentre, descriptive, longitudinal study was conducted over a three-year period following participants' arrival in France. The study design was ambispective, comprising two observation time points: an initial consultation within the first year after arrival in France, for which data were retrospectively retrieved from existing medical records, and a follow-up consultation, for which data were collected prospectively.

### 2.2 Setting, centres and study period

The study was conducted in hospitals providing specialised consultations for newly arrived migrant children in the Paris region, France. Three hospitals of Assistance Publique – Hôpitaux de Paris (AP-HP) agreed to participate: Robert-Debré Hospital (Paris), Necker-Enfants Malades Hospital (Paris), and Louis-Mourier Hospital (Colombes).

Initial consultations after arrival had taken place between January 1, 2018 and January 1, 2021. Follow-up consultations were conducted between February 1, 2022 and October 10, 2022.

### 2.3 Participants and study size

Migrant children were defined as children born abroad who arrived in France to settle permanently. Only accompanied minors were included, as unaccompanied minors are systematically relocated throughout France after their arrival, hindering efforts to reach them for follow-up.

The parents of accompanied international migrant children aged < 16 years who attended an initial consultation at one of the three study centres were contacted by telephone between January 1, 2022 and August 31, 2022 and invited to revisit the clinics for a follow-up consultation.

After obtaining parental consent, only the children who attended the follow-up consultation were included in this study.

As no prior assumptions could be made regarding the extent of change in health status following arrival, the sample size was not predetermined but convenience-based.

### 2.4 Follow-up procedures

For each child, one follow-up consultation was scheduled 24 to 40 months after the initial consultation. The time since arrival was defined as the interval between arrival in France and the initial consultation. This interval was calculated from the medical records and verified with the parents during the follow-up consultation. All follow-up consultations were conducted by the same clinical investigator during her final year of paediatric internship, under the supervision of a senior paediatrician. Data collection was standardised across the three centres, and comprised a structured health questionnaire, a clinical examination including anthropometric measurements, a vaccination review, and a psychomotor development assessment for children under 6 years of age.

### 2.5 Variables and measurements

Data from patients' medical records were retrieved using either Orbis® software or paper files. The data were recorded using Epidata® software. Missing data were recorded as such and handled without imputation.

The following information was collected retrospectively for the initial consultation and prospectively at the follow-up consultation by the same investigator: socio-demographic data available in medical records and reported by parents (date of birth, date of arrival in France, gender, region of birth according to the World Health Organisation (WHO)’s definition, type of housing, school enrolment and healthcare setting); vaccination status according to current French recommendations (Ministère des Solidarités et de la Santé, 2022), retrieved from child’s vaccination record; medical history reported by parents; pubertal stage assessed by the clinician during the consultation according to Tanner's classification (Marshall and Tanner, 1969, Marshall and Tanner, 1970); weight and height. Body weight was measured using a medical scale (Seca gmbh and Co., Germany) with an accuracy of ±100–200 g, and height using a stadiometer with an accuracy of ±0.5 cm. All scales were calibrated using a standardised 1 kg weight. Height and weight were used to calculate body mass index (BMI) according to the formula: BMI = weight (kg) / height (m^2^). BMI z-score was subsequently calculated. Stunting was defined as height-for-age < −2 SD, and thinness as BMI < −2 SD according to WHO standards (WHO Multicentre Growth Reference Study Group, 2006; Ministère des Solidarités et de la Santé, 2022). Overweight was defined as BMI > the International Obesity Task Force-25 (IOTF-25). Obesity was defined as BMI > IOTF-30. For children 〈 5 years of age, WHO Child Growth Standards curves were also used: wasting was defined as weight-for-height < −2 SD, overweight as weight-for-height 〉 +2 SD, and obesity as weight-for-height > +3 SD.

The following information was collected only at the follow-up consultation: for children < 6 years of age, parents completed the brief Child Development Inventory (CDI) questionnaire (Duyme et al., 2011) to assess psychomotor development. The Denver scale (Frankenburg et al., 1992) was also used for a parallel assessment. Delayed psychomotor development was defined as CDI development quotient < 85. For children > 6 years of age, any diagnosis of autism spectrum disorder (ASD) or attention deficit/hyperactivity disorder (ADHD) made since the initial consultation was retrieved from medical records or reported by parents.

### 2.6 Statistical analysis

Categorical variables are expressed as counts (percentages). Continuous variables are expressed as the means and standard deviations (SDs) for normally distributed variables and as medians and interquartile ranges (IQRs) for non-normally distributed variables. Univariate analyses were performed using Fisher's exact test for categorical variables and the Wilcoxon signed-rank test for continuous variables. Repeated measures within the same individuals were compared between the initial and follow-up consultations. Statistical analyses of changes in BMI were performed using the McNemar test for paired categorical data. Missing data, accounting for less than 5% of the total sample, were excluded from these analyses. A *p*-value < 0.05 was considered statistically significant. Statistical comparisons were performed using R® software (http://www.R-project.org).

### 2.7 Ethics

This study was performed in line with the principles of the Declaration of Helsinki. It was approved by the institutional ethics committee of Robert-Debré hospital (IRB 00,006,477). Parental informed consent was obtained for all participants included in the study, in accordance with the standards of the ethics committee.

## 3 Results

### 3.1 Participants

**Fig. 1:**
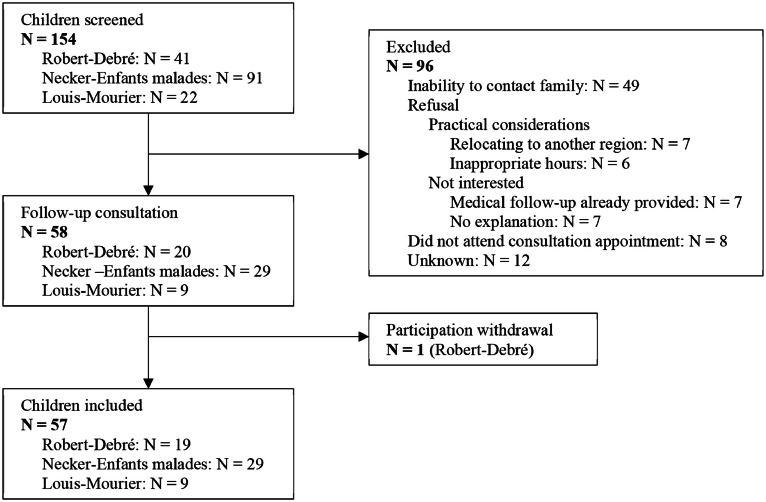
Flow chart.

A total of 154 migrant children accompanied by their parents attended specialised initial consultations for migrant children at Necker-Enfants malades (*N* = 91), Robert-Debré (*N* = 41) and Louis-Mourier (*N* = 22) hospitals between January 1, 2018, and January 1, 2021. All were invited for a follow-up consultation. Overall, 57 children (37% of the initial cohort and 54% of those reached by telephone) attended follow-up consultations between February 1, 2022 and October 10, 2022 and were included in the study. Main reasons for non-inclusion were the inability to reach the families of these children (*N* = 49) or refusal of the families to attend the follow-up consultation (*N* = 27) (Fig. 1).

Except for gender and region of origin, no major differences were observed between the socio-demographic characteristics of the 97 children who did not attend follow-up consultation, compared with the 57 children who did. The children from Africa predominated among the children who attended the follow-up consultation compared to children arriving from Europe or the Eastern Mediterranean region (Appendix A). The subgroups were too small to perform statistical comparisons.

### 3.2 Socio-demographic characteristics

The sex distribution was 24 males (42%) and 33 females (58%). The majority of children (*N* = 50, 88%) came from Africa, mainly West Africa and Central Africa (Table 1). All the families were French-speaking. The median age at arrival was 5.5 years (IQR 2.7–8.3), and the median age at the initial consultation was 5.9 years (IQR 3.1–8.9). The median delay between the arrival and the initial consultation was 3.6 months (IQR 2.2–5.6). The median delay between the initial and follow-up consultations was 2.8 years (IQR 2.2–3.3). The median age at follow-up consultation was 8.8 years (IQR 6–11.2)

**Table 1:** Birth regions.

	*N* = 57	
	N	%
Africa	50	88%
North Africa	2	4%
West Africa	34	60%
Central Africa	11	19%
East Africa	3	5%
Europe	1	2%
Eastern Mediterranean	5	9%
Americas	1	2%

Compared with the initial consultation where 7 families were homeless, all the families were housed at the time of the follow-up visit. However, 26 (46%) remained in unstable housing (social hotels or hostels) (Table 2).

**Table 2:** Social characteristics.

	Initial consultation		Follow-up consultation	
	*N* = 57		*N* = 57	
	N	%	N	%
**Housing type**				
Personal housing (occupied by a single family)	14	25%	28	49%
Lodging with a family or acquaintance	3	5%	3	5%
Social hotel	14	25%	17	30%
Hostel or shared space with other families	11	19%	9	16%
Homeless	7	12%	0	-
Missing data	8	14%	0	-
**School attendance**				
Yes	16	28%	55	96%
No	26	46%	0	-
Not applicable (< 3 years of age)	12	21%	2	4%
Missing data	3	5%	0	-
**Medical follow**				
Pediatrician or general practitioner	10	18%	22	38%
Intrahospital consultations only	0	-	2	4%
No medical follow-up	46	81%	33	58%
Missing data	1	2%	0	-
**Vaccination status**				
Complete	11	19%	45	79%
Incomplete	44	77%	7	12%
Missing data	2	4%	5	9%

All the school-aged children attended school at the time of the follow-up visit.

### 3.3 Medical follow-up since the initial consultation

Since their initial consultation, 24/57 children (42%) had received medical follow-up.

Six children (11%) had presented with chronic underlying conditions upon arrival, including epilepsy, motor disability, or heart disease. Two of these children were no longer receiving medical care at the time of the follow-up consultation.

Seven of the 44 children with incomplete vaccination at the initial consultation had not completed their vaccination schedule at the follow-up visit (Table 2).

### 3.4 Evolution of anthropometric status since the initial consultation

Changes in anthropometric status between the initial and follow-up consultations were assessed. One non-ambulatory patient with a physical disability was excluded due to missing height data. Mean BMI was significantly higher at the follow-up visit compared to the initial visit (0.37 and 0.04 SDS respectively, *p <* *0.01)*, and the prevalence of overweight was significantly higher at the follow-up visit (11 (19%) and 3 children (5%), respectively, *p <* *0.01*) (Table 3). No significant difference was observed in the prevalence of obesity or thinness between the initial and follow-up consultations.

**Table 3:** Anthropometric status, development quotient for children < 6 years.

	Initial consultation *N* = 57		Follow-up consultation		
	N	%	N	%	p
**Anthropometric status**			*N* = 57		
Obesity	1	2%	3	5%	0.0545
Overweight	3	5%	11	19%	**0.0063**
Thinness	1	2%	4	7%	0.0545
Stunting	2	4%	1	2%	
Missing data	1	2%	1	2%	
**Children < 6 years of age: development quotient**			*N* = 14		
Developmental delay (DQ < 85)	-		2	14%	-
Normal development	-		11	79%	-
Missing data	-		1	7%	-

### 3.5 Psychomotor and pubertal development

Developmental test results using the Brief CDI scale were normal for 11 of the 14 children (79%) tested at the follow-up consultation. Two children (14%) not previously recognised as having developmental delay were identified with this condition (Table 3). These results were consistent with the assessments made using the Denver development scale. No diagnosis of ASD or ADHD was reported by parents.

Moreover, no early puberty was observed at the follow-up consultation.

## 4 Discussion

Very few paediatric studies have evaluated the long-term health outcomes of first-generation international migrant children after arrival in a high-income country (Gualdi-Russo et al., 2014; Jaeger et al., 2012; Salami et al., 2021; Salami et al., 2022). In our study many findings suggest that the precarity of this population still impacts child health status more than two years after arrival.

While no families were homeless, nearly half of the children still had unstable housing more than two years after arrival. Such living conditions may be associated with overcrowding, cold and dampness, poor ventilation and poor hygiene. A recent systematic review assessing housing inequalities and health outcomes among migrants and refugees in high-income countries, reported that poor housing was consistently associated with adverse physical and mental health outcomes, including respiratory illnesses as well as anxiety and depression (Rana et al., 2025).

Furthermore, only 42% of the children had medical follow-up with a general practitioner or a paediatrician since their initial visit to the specialised migrant clinic. Thus, although all the children had been vaccinated on arrival according to the national recommendations, 12% had not received the recommended boosters. These findings are particularly worrying as they reflect a persistent inequity of access to the healthcare system despite an extended length of stay. In France, primary care for migrant children — including general practitioner visits and mandatory vaccinations — is covered by public funding through two distinct schemes: the national health insurance system, which includes the Couverture Santé Solidaire (CSS) for people with limited resources, and the Aide Médicale d'État (AME), directly funded by the state for children of undocumented migrants. Moreover, several health centres, such as maternal and child health centres for children under 6 years of age, or urban ambulatory health centres offer care without requiring out-of-pocket payments. However, many families reported difficulties in identifying a general practitioner, reflecting a lack of understanding of the healthcare system. As outlined in a 2017 report by the French Academy of Medicine (Spira, 2017), although there are several associations dedicated to facilitating access to healthcare for the most vulnerable people, this access remains limited due to considerable coordination difficulties between healthcare facilities. The same report also indicates lower vaccination coverage rates among children from the most disadvantaged families. Language represents another significant barrier to healthcare access. This has been illustrated, for instance, by studies on the monitoring of pregnant women in France (Eslier et al., 2023): women with poor French language skills received suboptimal prenatal care. Finally, discriminatory attitudes among healthcare personnel themselves may further compound these difficulties. Explicit discrimination has been documented, with several reports describing doctors refusing to treat patients covered by CSS or AME (Le Rolland et al., 2023). More subtle forms of bias have also been suggested: foreign-born mothers have been shown to receive less adequate information regarding trisomy 21 screening (Anselem et al., 2021). Improving support throughout the care pathway appears essential. This includes providing targeted health information and strengthening coordination between dedicated consultations upon arrival and subsequent referrals to community physicians.

One of the study's key findings was the substantial rise in the proportion of overweight children in the first two years after arriving in France, increasing from 5% to 19% (*p* < 0.01). The 3 to 5% increase of the obesity prevalence was not significant. After two years in France, the overweight and obesity status of these migrant children was comparable to that of native French children. A Santé Publique France study in 2015 reported a prevalence of overweight children of 17%, including 3.9% of children with obesity (Verdot et al., 2017). According to this report, the prevalence of being overweight or obese was similar in all age groups. Therefore, in this migrant population, the increase of overweight between the two consultations cannot be attributed to the ageing of the study population alone. Studies conducted in European countries have found wide variations in overweight and obesity prevalence rates between migrant and native-born children. Overweight rates ranged from 8.9% to 37.5% in migrant children and 6.4% to 27.3% in native-born children. Obesity rates ranged from 1.2% to 15.4% in migrant children and 0.6% to 11.6% in native-born children (Gualdi-Russo et al., 2014; Labree et al., 2011). In a study of first-generation migrants aged 12–19 years in Canada, the z-score for body mass index (zBMI) increased by 0.02 for young migrants each year in Canada (Wahi et al., 2014). The increase observed in our study seemed faster and particularly concerning.

Some evidence suggests that populations from certain geographic areas may be genetically susceptible to becoming overweight. Indeed, the tendency to be overweight is more pronounced among migrants from Africa who represent 88% of the children in our study, as well as those from Middle East and Latin America and less pronounced among those from Asia (Gualdi-Russo et al., 2014; Labree et al., 2011; Menigoz et al., 2016). We have not studied the changes in lifestyle habits of families since their arrival in France and therefore cannot estimate the role of acculturation in weight gain. However, this finding may be related to persistent instability even after several years in France, whereby financial constraints and housing issues, rather than food preferences, often limit access to adequate nutrition. This is consistent with several studies showing that food insecurity is associated with overweight or obesity in children (St Pierre et al., 2022). For example, Spanish children in food-insecure households had increased relative risks of overweight (RR 2.41) or obesity (RR 1.99) compared to children in food-secure households (Ortiz-Marrón et al., 2022).

Therefore, the increase in overweight status among the migrant children suggests the need for specific support for their families, particularly provision of information on nutritional needs, as general public campaigns are likely less accessible to and less culturally and linguistically appropriate for this population. Although educational programs would not eliminate the economic impediments leading to poor nutrition, perhaps such interventions could minimise this problem.

In this study we also evaluated developmental aspects as puberty and neurocognitive status in the follow-up consultation. There was no evidence that migrant children are at an increased risk of early puberty; however the sample size was small as nearly half of the participants were above the age threshold for early puberty upon arrival in France. An increased risk of central precocious puberty in internationally adopted children compared to children in the host country has been well described (Soriano-Guillén et al., 2010; Teilmann et al., 2006), with early puberty rates in girls ranging from 30% to 44.9% (Mason and Narad, 2005). This phenomenon is less well documented in international migrant children: some studies find faster pubertal development in migrant children compared to natives of the arrival country or to non-migrant children who remained in their country of origin (Gualdi-Russo et al., 2014). Other studies find no increased risk (Soriano-Guillén et al., 2010; Teilmann et al., 2006).

In the present study, two children presented with delayed psychomotor development, which had not been detected prior to the follow-up consultation. Although the numbers were small, migrant children may be at increased risk of developmental delays. For example, a study of poorly housed children in precarious situations categorised 80.9% as having moderate to low adaptive functioning according to the second edition of the Vineland Adaptive Behaviour Scale (Darbeda et al., 2018). Repeated assessments of psychomotor development of migrants appear to be warranted, especially if language and poor medical follow-up after arrival lead to missed opportunities to detect delayed psychomotor development.

The main strength of our study was its longitudinal design*,* which enabled us to observe the changes in children's health following an extended stay in France. Furthermore, the follow-up consultation data were obtained in a consistent manner, as the evaluator was the same for all three centres and the children were assessed systematically using the same protocol.

The main weakness is the small sample size and the high number of lost to follow-up children since the initial consultation, as only 37% of children among the eligible population attended the follow-up consultation. This may have introduced selection bias, although we did not observe any socio-demographic differences between children who were and were not included except for birth region and gender. The lack of a predetermined sample size may limit the representativeness of the study population and the external validity of the findings. The inclusion of only French-speaking families at follow-up consultations may reflect systematic difficulties in reaching non-French-speaking families. These families are likely to be less socially integrated and more socioeconomically disadvantaged, suggesting that our findings may underestimate the true extent of poor health outcomes among migrant children. The reasons for the high proportion of children born in sub-Saharan Africa who participated in the follow-up visit, compared to those who did not remain unclear. However, due to these limitations, our results have limited generalizability to broader migrant populations, particularly those originating from Europe and Asia, who are underrepresented in this study. Our study did not include the health status of unaccompanied migrant children, but focused specifically on migrant children who travelled with their parents to France. Moreover, the collection of diagnoses of ASD or ADHD was partly based on parents' statements, which may have led to recall bias. Finally, this study was descriptive without a comparison population, which also limits the strength of the findings.

## 5 Conclusion

In this study, migrant children continued to experience precarious living conditions at least two years after arrival in France. More than 50% have received no medical follow-up despite the wide availability of free medical care. Nutritional issues, such as the rapid increase in overweight, are particularly concerning and may be associated with precarious housing. Any physician consulting with children who have recently migrated should ensure that they receive medical follow-up or refer their families accordingly. Alongside the efforts made to welcome migrant children, these observations should lead to improved long-term support for families in terms of social and medical care, as well as information, health education and prevention. These measures should be targeted and adapted to this specific and vulnerable population. Studies conducted on larger populations and with longer follow-up would enable to better understand and address the obstacles to improving the health of migrant children arriving in high-income countries. Lifestyle factors associated with weight gain in this population deserve particular attention.

## Funding

This research did not receive any specific grant from funding agencies in the public, commercial, or not-for-profit sectors.

## CRediT authorship contribution statement

**Alice Grognet Essaian:** Writing – original draft, Investigation, Formal analysis, Conceptualization. **Frédéric Sorge:** Writing – review & editing, Resources, Conceptualization. **Enora Le Roux:** Writing – review & editing, Formal analysis. **Sophie Guilmin-Crépon:** Writing – review & editing, Formal analysis. **Juliette Goutines:** Writing – review & editing. **Laurie C. Miller:** Writing – review & editing. **Marion Taine:** Writing – review & editing. **Romain Basmaci:** Writing – review & editing, Resources, Conceptualization. **Albert Faye:** Writing – review & editing, Supervision, Resources, Conceptualization.

## Declaration of competing interest

The authors declare that they have no known competing financial interests or personal relationships that could have appeared to influence the work reported in this paper.

## Figures and Tables

**Table:** 

	Included		Non-included	
	*N* = 57		*N* = 97	
	N	%	N	%
Sex				
Female	33	58%	46	47%
Male	24	42%	51	53%
Birth region				
Africa	50	88%	52	54%
Europe	1	2%	24	25%
Eastern Mediterranean	5	9%	16	16%
South-East Asia	0	-	4	4%
Americas	1	2%	1	1%
Age				
0 to < 3 years	14	25%	30	31%
3 to < 6 years	16	28%	31	32%
6 to < 11 years	16	28%	22	23%
11 years and more	11	19%	14	14%
Housing type				
Personal housing (occupied by a single family)	14	25%	14	14%
Lodging with a family or acquaintance	3	5%	6	6%
Social hotel	14	25%	16	16%
Hostel or shared space with other families	11	19%	30	31%
Homeless	7	12%	0	-
Missing data	8	14%	31	32%
Medical history				
Chronic pathology	6	11%	14	14%
Missing data	0	-	10	10%
